# TRPV4-linked oxidative–lysosomal stress in myocardial ischemia/reperfusion: disease-matched transcriptomics and functional analysis in OGD/R cardiomyoblasts

**DOI:** 10.3389/fcell.2026.1945533

**Published:** 2026-09-29

**Authors:** Jie Yang, Jincheng Li, Guanyao Li, Heyu Zhong, Hang Yang, Kai Zhong, Fan Wang, Zhou Huang, Dongling Huang, Wanxia Wei, Xueling Lu, Guodong Pang, Yuqinlin Chen, Yiling Zhai, Wei Wang

**Affiliations:** 1 Department of Emergency, The First Affiliated Hospital of Guangxi Medical University, Nanning, Guangxi, China; 2 Department of Emergency Medicine, Longgang Central Hospital of Shenzhen, Shenzhen, Guangdong, China; 3 College of Orthopedics & Traumatology, Guangxi University of Chinese Medicine, Nanning, Guangxi, China; 4 Guangxi University Key Laboratory of Emergency Medicine, The First Affiliated Hospital of Guangxi Medical University, Nanning, Guangxi, China

**Keywords:** autophagy, bulk RNA-seq, lysosomal membrane permeabilization, microarray, myocardial ischemia/reperfusion injury, OGD/R, oxidative stress, TRPV4

## Abstract

**Background:**

Myocardial ischemia/reperfusion (MI/R) injury involves oxidative stress, lysosomal dysfunction, and altered autophagy. TRPV4 may link Ca^2+^ dysregulation to these processes, but its role in lysosomal membrane permeabilization (LMP) and autophagic impairment remains unclear.

**Methods:**

Three disease-matched mouse-heart GEO cohorts (GSE281940, GSE130217, and GSE160516) were analyzed using limma-based differential expression, prespecified oxidative, lysosomal, and autophagy-related gene sets, cross-cohort comparisons, and within-cohort correlations. H9C2 cardiomyoblasts subjected to OGD/R were used to examine intracellular Ca^2+^, ROS, LMP, autophagy-related changes, inflammatory cytokine release, and cell injury after pharmacological modulation of TRPV4 and ROS.

**Results:**

All three cohorts showed clear MI/R-associated transcriptional changes. Hmox1 was consistently increased across all five prespecified contrasts, whereas *Trpv4* showed modest, time-dependent changes without reaching FDR-adjusted significance. *Trpv4* was positively correlated with *Map1lc3b* in all three cohorts (Spearman ρ = 0.73–0.83, nominal P < 0.05), while associations with oxidative and lysosomal genes were more variable. In H9C2 cardiomyoblasts, OGD/R increased TRPV4 mRNA expression, intracellular Ca^2+^, and ROS levels. TRPV4 inhibition reduced ROS accumulation, attenuated LMP-related changes, and decreased LC3B and p62 accumulation, whereas TRPV4 activation produced the opposite effects. NAC attenuated TRPV4 activation-induced ROS and lysosomal injury, while t-BHP partially reversed the effects of TRPV4 inhibition. OGD/R also increased IL-1β, IL-6, and TNF-α release, which was reduced by TRPV4 inhibition.

**Conclusion:**

The transcriptomic data indicate a context-dependent TRPV4 response within a conserved MI/R stress background. The H9C2 cardiomyoblasts experiments support the involvement of the TRPV4–ROS–LMP pathway in lysosomal injury, impaired autophagic degradation, inflammatory responses, and OGD/R-induced cardiomyoblast injury.

## 1 Introduction

Acute myocardial infarction and the resultant myocardial ischemia/reperfusion (MI/R) injury are characterized by Ca^2+^ dysregulation, reactive oxygen species (ROS) accumulation, sterile inflammation, and interacting regulated cell-death programs that shape cardiomyocyte fate (Francisco and Del, 2023; He et al., 2022; Bugg et al., 2020). Rather than reflecting a single death mechanism, MI/R engages overlapping apoptotic, necrotic, inflammatory, and autophagy-associated responses across ischemia and reperfusion.

Transient receptor potential vanilloid 4 (TRPV4) is a non-selective Ca^2+^-permeable cation channel expressed in cardiovascular tissues and involved in intracellular Ca^2+^ homeostasis (Chaigne et al., 2023). Previous studies have implicated TRPV4 in myocardial ischemia/reperfusion injury, showing that excessive TRPV4 activation can increase Ca^2+^ influx and aggravate cardiomyocyte injury (Wu et al., 2017; Dong et al., 2025). TRPV4 activation has also been associated with oxidative stress and autophagy-related changes in cardiac cells (Sun et al., 2022; Ma et al., 2024). These findings suggest that TRPV4 may act upstream of several stress responses during reperfusion, but the downstream events linking TRPV4-associated Ca^2+^ dysregulation to lysosomal and autophagic dysfunction remain unclear.

ROS may provide an important link between TRPV4 activation and autophagy-related dysfunction during reperfusion. Autophagy plays a context-dependent role in MI/R injury: moderate activation may help remove damaged organelles and maintain cellular homeostasis, whereas excessive or impaired autophagy during reperfusion can contribute to further injury (Aljakna Khan and Sabatasso, 2025). Reperfusion-associated ROS production has been closely linked to changes in autophagic activity, and previous studies have shown that excessive ROS can disturb autophagic homeostasis and impair the normal turnover of damaged cellular components (Peng et al., 2024; Yi et al., 2020; Sun et al., 2023). TRPV4-mediated Ca^2+^ entry has also been reported to enhance oxidative stress (Sun et al., 2022), suggesting that TRPV4 may contribute to autophagy-related abnormalities through increased ROS production. However, the mechanism by which TRPV4-associated ROS affects the downstream autophagy–lysosome system remains unclear.

Lysosomal membrane permeabilization (LMP) may represent an important link between oxidative stress and defective autophagic degradation. Excessive ROS can compromise lysosomal membrane integrity and promote the redistribution of lysosomal cathepsins into the cytoplasm (Chang et al., 2022; Qi et al., 2024). Because lysosomes are required for the terminal degradation step of autophagy, LMP can interfere with autophagic flux and promote the accumulation of autophagic substrates. Lysosomal damage has also been linked to inflammatory signaling in several pathological settings, where lysosomal destabilization and cathepsin release can promote inflammasome activation and cytokine production (Zhang et al., 2019; Wu et al., 2022). These findings suggest a possible connection among oxidative stress, autophagy–lysosome dysfunction, and inflammation.

In the present study, we combined three disease-matched mouse-heart transcriptomic cohorts with H9C2 OGD/R experiments. The transcriptomic analysis characterized time-dependent oxidative, lysosomal, and autophagy-associated responses and evaluated TRPV4 as an association-level bridge across cohorts. Guided by these expression patterns, pharmacological perturbation and rescue experiments tested whether TRPV4-sensitive calcium and ROS responses were linked to LMP and impaired autophagic flux.

## 2 Materials and methods

### 2.1 Transcriptomic analysis

#### 2.1.1 Dataset acquisition and differential expression analysis

Three mouse-heart GEO cohorts were selected after sample-level review. GSE281940 included sham hearts and hearts subjected to 30 min of ischemia followed by 2 h of reperfusion. GSE130217 included left-ventricular samples from young wild-type mice subjected to sham surgery or 45 min of LAD occlusion followed by 24 h of reperfusion. GSE160516 included sham hearts and hearts collected after 6, 24, or 72 h of reperfusion following 30 min of ischemia. For the RNA-seq datasets, genes with counts per million (CPM) ≥ 1 in at least three samples were retained. Library-size normalization was performed using the trimmed mean of M-values (TMM) method, followed by voom transformation and differential expression analysis with the limma package. For GSE160516, expression values from the Clariom S Mouse platform were mapped to mouse gene symbols using the clariomsmousetranscriptcluster. db annotation package (version 8.8.0), and differential expression was assessed with limma. P values were adjusted using the Benjamini–Hochberg method, and FDR <0.05 was considered statistically significant.

### 2.2 Cross-cohort analysis of prespecified stress, lysosomal, and autophagy gene sets

The cohorts were not merged into a single expression matrix. Within each study, normalized expression was used to calculate prespecified oxidative-response (*Hmox1*, *Cybb*, *Nox4*), lysosome (*Atp6v1h*, *Lamp1*, *Lamp2*, *Ctsb*, *Ctsd*), and autophagy (*Map1lc3b*, *Sqstm1*, *Becn1*) module scores. *Trrpv4* effects were compared across the five prespecified MI/R contrasts, and within-cohort Spearman correlations with axis genes were reported as descriptive, nominal associations.

### 2.3 Time-resolved microarray and cross-platform concordance analysis

GSE160516 was analyzed separately at 6, 24, and 72 h of reperfusion, with each time point compared with sham using limma. Time-dependent changes in Trpv4 and selected oxidative-response, lysosomal, and autophagy-related genes were then examined. Cross-platform concordance was assessed using Spearman correlation of gene-level log2 fold changes among genes shared between the compared datasets. The early comparison was performed between GSE281940 2 h reperfusion and GSE160516 6 h reperfusion, whereas the 24 h comparison was performed between GSE130217 and the 24 h contrast of GSE160516. Correlations were calculated using the intersecting gene set available in both datasets.

### 2.4 Within-cohort correlation analysis

Within each cohort, Spearman correlations were calculated between *Trpv4* expression and the prespecified oxidative-response, lysosomal, and autophagy-related genes. Correlations were analyzed separately for each dataset and were not pooled across studies. For GSE160516, correlations were calculated across the complete time-course cohort and were interpreted as descriptive within-cohort co-variation rather than evidence of direct gene regulation. Because these analyses were exploratory, nominal P values are reported.

## 3 Cell biology experiments

### 3.1 Reagents

HC-067047 (Cat. No. HY-100208, MCE, USA); GSK1016790A (Cat. No. HY-19608, MCE, USA); NAC (Cat. No. HY-B0215, MCE, USA); LLOMe (Cat. No. HY-101680, MCE, USA); CQ (Cat. No. HY-17589, MCE, USA) were from MedChemExpress. Mannose and t-BHP were from Sigma-Aldrich (3458-28-4, 75-91-2).

### 3.2 Cell culture and OGD/R model

Rat H9C2 cardiomyoblasts (CL-0089, Procell, China) were cultured in high-glucose DMEM at 37 °C in 5% CO_2_. For OGD/R, cells were switched to glucose/serum-depleted DMEM in 95% N_2_/5% CO_2_ for 6 h, then returned to standard medium and normoxia for the designated reoxygenation period.

### 3.3 Cell treatment

HC-067047 (500 nM), GSK1016790A (300 nM), NAC (5 mM), or mannose (10 mM) was added at the beginning of reoxygenation. t-BHP (150 μM), LLOMe (300 μM), and CQ (30 μM) were added during the final 2 h of reoxygenation. Equal volumes of vehicle were added to control groups.

### 3.4 Cell viability and LDH assay

Cell viability was assessed by CCK-8 assay. LDH enzymatic activity was measured using a commercial kit (A020-2-2, Jiancheng, China) as an index of membrane injury.

### 3.5 Ca^2+^, ROS, LysoTracker, and immunofluorescence

Intracellular Ca^2+^ levels were measured using the Fluo-4 AM probe (S1060, Beyotime, China). Intracellular ROS was detected by DCFH-DA (S0033S, Beyotime, China). Lysosomal integrity was assessed by LysoTracker Red (C1046, Beyotime, China). For immunofluorescence, cells were fixed in 4% paraformaldhyde, permeabilized with 0.25% Triton X-100, and incubated with anti-CTSD (1:200; AF5191, Affinity, China), anti-p62 (1:200; A19700, ABclonal, China) or anti-LC3B (1:200; A19665, ABclonal, China) antibodies, then with Cy3-conjugated secondary antibody. Images were acquired on a Nikon Eclipse C1 fluorescence microscope.

### 3.6 Measurement of inflammatory cytokines by ELISA

The concentrations of IL-1β, IL-6, and TNF-α in the culture supernatant were measured using rat-specific ELISA kits for IL-1β (BMS630), IL-6 (BMS625), and TNF-α (BMS622) (Invitrogen, Thermo Fisher Scientific, Waltham, MA, USA), according to the manufacturer’s instructions. After the indicated treatments, culture supernatants were collected and centrifuged to remove cellular debris. Samples and standards were assayed in duplicate, and absorbance was measured at 450 nm using a microplate reader. Cytokine concentrations were calculated from the corresponding standard curves and expressed as pg/mL.

### 3.7 Quantitative real-time PCR (qRT-PCR)

Total RNA was extracted from H9C2 Cardiomyoblasts using the FreeZol Reagent Kit (R711-01, Vazyme Biotech, China). Complementary DNA (cDNA) was synthesized using a reverse transcription kit (R323-01, Vazyme Biotech, China). qRT-PCR was performed with ChamQ Universal SYBR qPCR Master Mix (Q711-02, Vazyme Biotech, China). β-Actin served as the internal reference gene, and relative mRNA expression levels were calculated using the comparative threshold cycle (2^−ΔΔCT^) method. The sequences of the primers used for amplification (Sangon Biotechnology, China) are as follows:

TRPV4 Forward primer (5’ > 3′) AAG​AAG​CGC​CTG​ACT​GAT​G;
Reverse primer (5’ > 3′) CAC​TGG​GAT​GGT​GTC​GTT​T.
β-actin Forward primer (5’ > 3′) CCT​CAC​TGT​CCA​CCT​TCC​A;
β-actin Reverse primer (5’ > 3′) GGG​TGT​AAA​ACG​CAG​CTC​A.

### 3.8 Statistical analysis

Data are presented as mean ± SD. Normality and homogeneity of variance were verified by Shapiro–Wilk and Levene’s tests. Multiple-group comparisons used one-way ANOVA; two-group comparisons used independent-samples t-test (SPSS v26.0). *P* < 0.05 was considered significant.

## 4 Results

### 4.1 Disease-matched transcriptomes define the MI/R response and characterize *Trpv4* expression

To establish the disease and tissue context, we first examined sample structure and quality in the three cohorts (Figure 1A). Sham and MI/R hearts separated by PCA in both bulk RNA-seq studies (Figures 1B,C). GSE281940 identified 1,619 genes with FDR <0.05 (532 upregulated and 1,087 downregulated), whereas GSE130217 identified 122 (101 upregulated and 21 downregulated; Figures 1D,E). *Hmox1* was consistently increased (GSE281940 log2FC = 1.48, FDR = 0.017; GSE130217 log2FC = 2.19, FDR = 0.007), and *Atp6v1h* was increased in GSE281940 (log2FC = 0.90, FDR = 0.0002). *Trpv4* showed modest positive fold changes in both RNA-seq cohorts, although neither reached FDR-adjusted significance (GSE281940: log2FC = 0.48, FDR = 0.212; GSE130217: log2FC = 0.39, FDR = 0.565; Figure 1F). Thus, both datasets showed a clear MI/R transcriptional response, whereas the change in *Trpv4* expression was modest and did not meet the predefined significance threshold.

**FIGURE 1:**
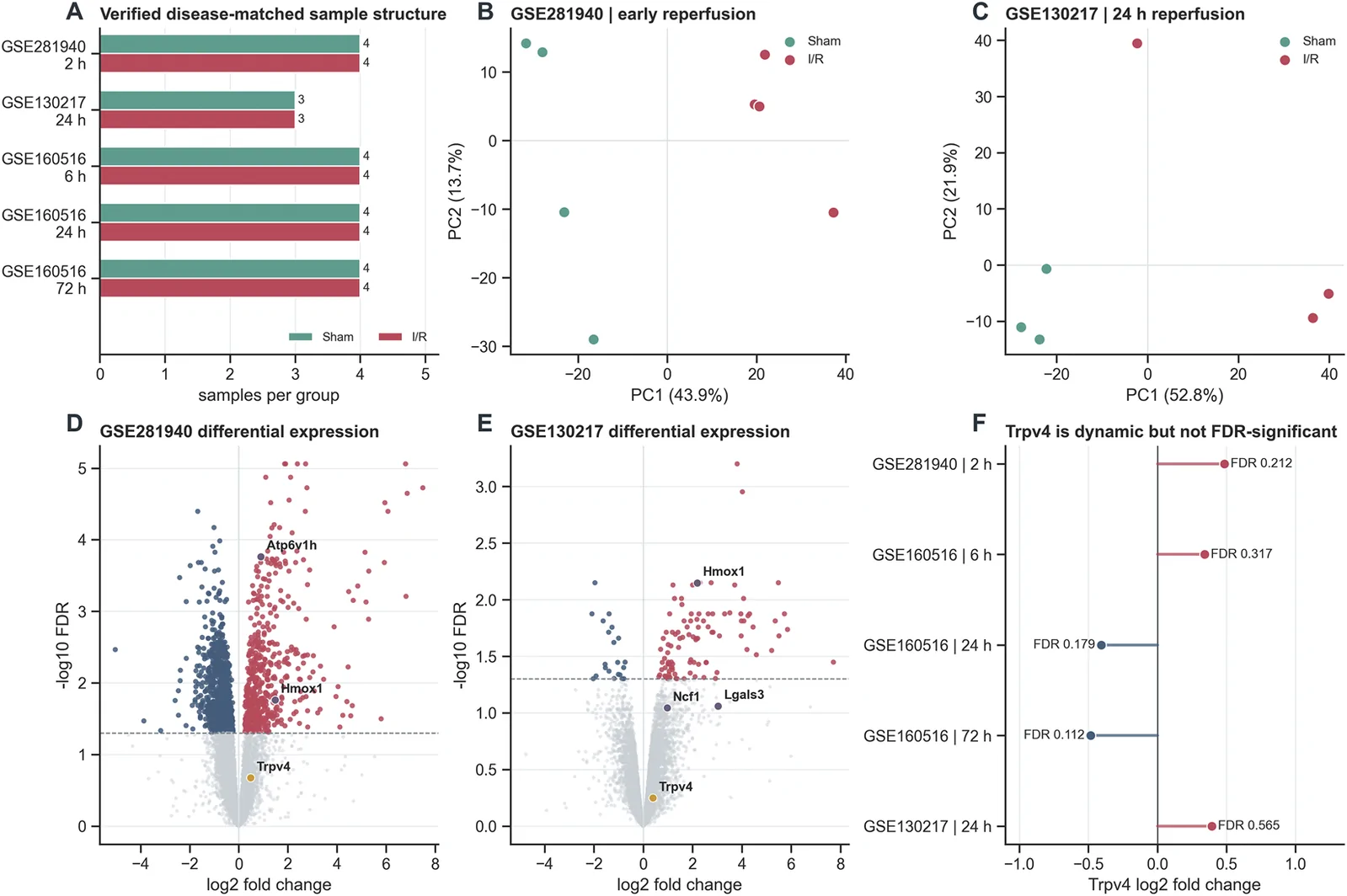
Disease-matched mouse-heart transcriptomes establish the MI/R response and characterize Trpv4 expression. **(A)** Sham and MI/R sample structure across the five prespecified contrasts from GSE281940, GSE130217, and GSE160516. **(B,C)** PCA of the two bulk RNA-seq cohorts, showing separation of sham and MI/R hearts. **(D,E)** RNA-seq volcano plots with *Hmox1*, *Atp6v1h*, and *Trpv4* labeled. **(F)** *Trpv4* log2 fold changes and FDR values across all five prespecified contrasts.

### 4.2 Time-resolved cardiac transcriptomics reveals persistent oxidative-stress-related and dynamic lysosomal/autophagy-related change

The GSE160516 time course separated sham, 6, 24, and 72 h reperfusion hearts by PCA and showed a broadening transcriptional response (4,397, 5,757, and 4,935 FDR<0.05 genes at 6, 24, and 72 h, respectively; Figure 2A). Hmox1 remained strongly increased at all three time points (log2FC = 6.20, 4.57, and 5.83; FDR = 6.2e-05, 5.6e-04, and 1.0e-04). Atp6v1h was increased at 6 and 24 h, and Ctsb, Ctsd, Lamp1, Lamp2, and Sqstm1 showed time-dependent changes, whereas Map1lc3b decreased at 24 h (Figure 2B). These patterns indicate a sustained oxidative-stress-related transcriptional response accompanied by dynamic changes in genes related to lysosomal function and autophagy during reperfusion (Figures 2C–F).

**FIGURE 2:**
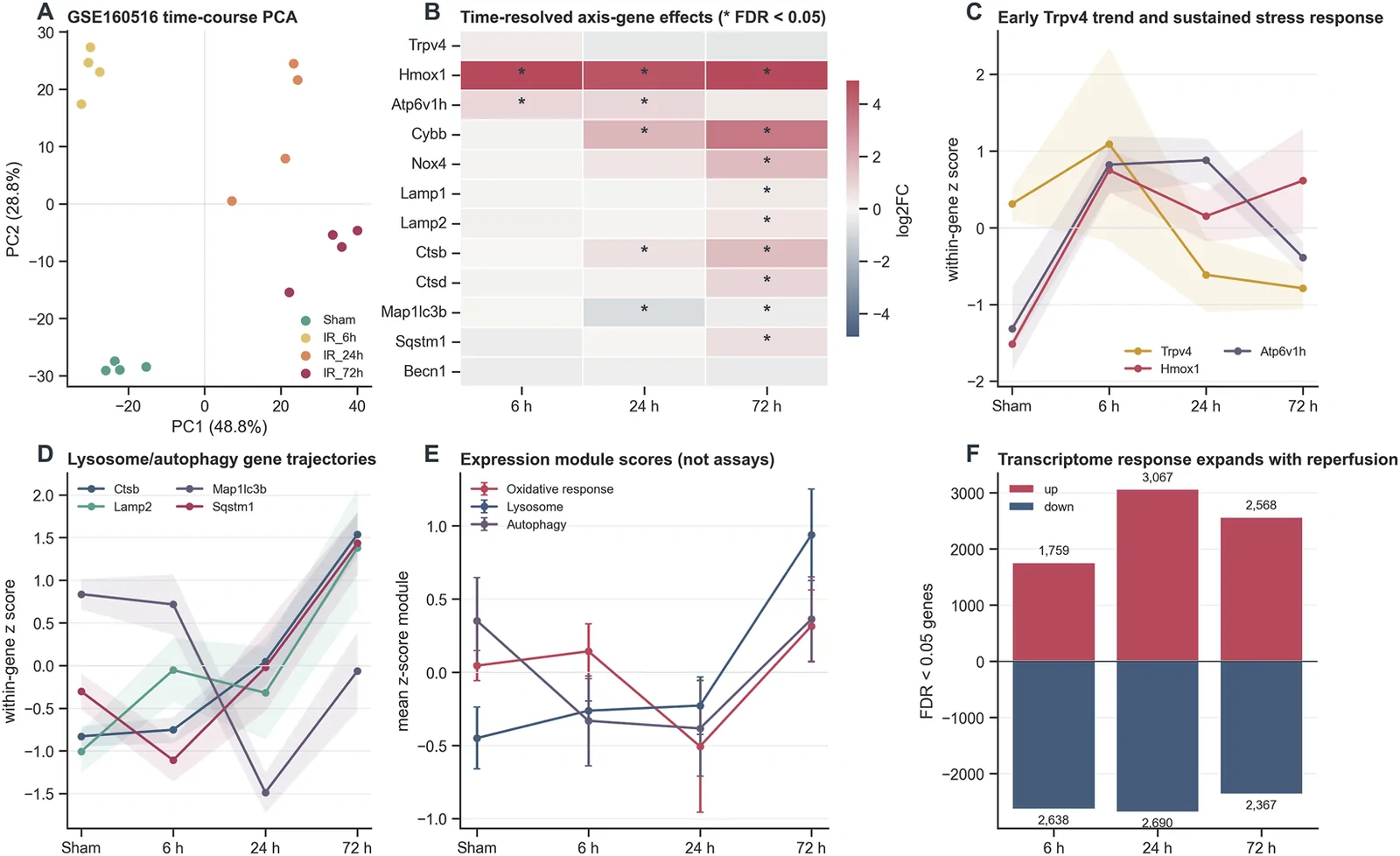
Time-resolved mouse-heart transcriptomics reveals persistent stress-related and dynamic lysosomal/autophagy-associated changes. **(A)** PCA of sham and 6, 24, and 72 h reperfusion groups in GSE160516. **(B)** Log2 fold changes of prespecified oxidative-response, lysosomal, and autophagy-related genes at each time point; asterisks denote FDR >0.05. **(C,D)** Within-cohort standardized trajectories for *Trpv4*, *Hmox1*, *Atp6v1h*, and selected lysosome/ autophagy genes. **(E)** Prespecified gene-set expression scores. **(F)** Numbers of up- and downregulated genes at each reperfusion time.

### 4.3 Cross-cohort comparison distinguishes conserved stress responses from context-dependent *Trpv4* expression

We next compared the five prespecified contrasts without pooling samples across studies. Hmox1 was positive and FDR-significant in all five contrasts, and Atp6v1h was positive in all five with FDR significance in three (Figure 3A, B). In contrast, Trpv4 showed positive fold changes in three contrasts and negative fold changes in two, without reaching FDR-adjusted significance in any comparison (Figures 3A,B). Genome-wide log2 fold-change concordance was modest at the early comparison (RNA-seq versus 6 h array, Spearman ρ = 0.33; Figure 3C) and stronger at 24 h (ρ = 0.63; Figure 3D). These results indicate that some stress-related transcriptional responses are conserved across datasets, whereas Trpv4 expression is more dependent on experimental context, reperfusion time, and platform (Figures 3E,F).

**FIGURE 3:**
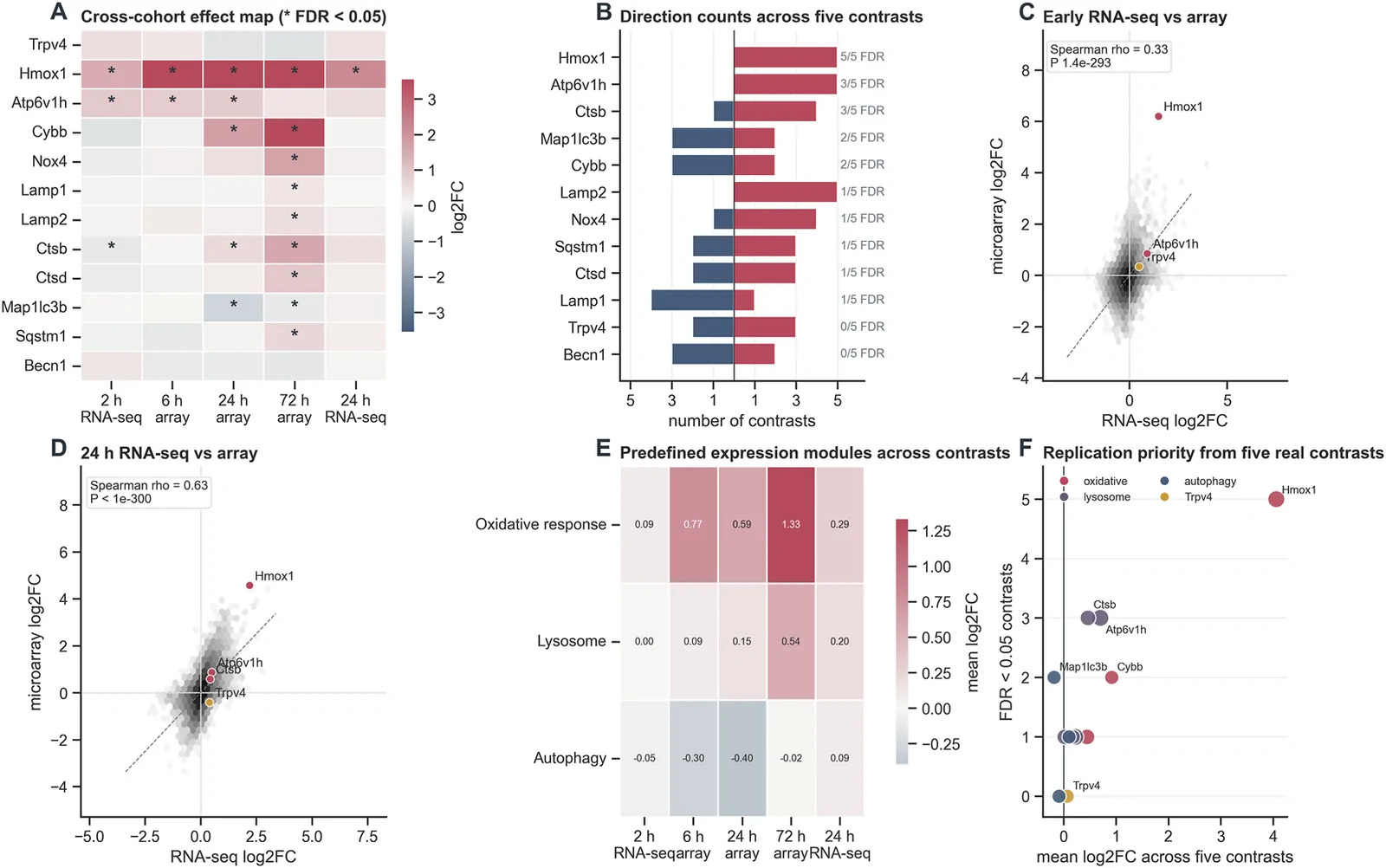
Cross-cohort comparison distinguishes conserved myocardial stress responses from context-dependent Trpv4 expression. **(A)** Effect-size map for prespecified oxidative, lysosomal, and autophagy genes across five disease-matched contrasts; asterisks denote FDR <0.05. **(B)** Counts of positive (red) and negative (blue) directions and FDR discoveries for each gene. **(C,D)** Genome-wide RNA-seq-versus-array concordance at early and 24 h reperfusion. **(E)** Mean log2 fold change across genes within each prespecified gene set. **(F)** Mean effect size, number of FDR-significant contrasts, and direction consistency for each prespecified gene across the five contrasts.

### 4.4 Within-cohort *Trpv4* associations with autophagy and oxidative-stress related genes

Finally, we assessed the relationships between *Trpv4* and selected genes related to autophagy, lysosomal function, and oxidative stress within each cohort (Figure 4A). *Map1lc3b* showed a positive correlation with *Trpv4* in all three datasets (GSE281940: ρ = 0.76, P = 0.028; GSE130217: ρ = 0.83, P = 0.042; GSE160516: ρ = 0.73, P = 0.0013; Figures 4B–D). *Becn1* showed a similar positive trend. By contrast, *Cybb* was negatively correlated with *Trpv4* in GSE281940 (ρ = −0.81, P = 0.015) and GSE160516 (ρ = −0.77, P = 0.00048), while correlations with lysosomal genes varied across datasets (Figures 4A,E). Across cohorts, *Map1lc3b* showed the most consistent positive relationship with *Trpv4*, whereas the associations with oxidative and lysosomal genes were less uniform (Figure 4F). These results provided a rationale for examining ROS, lysosomal integrity, and autophagy in the subsequent H9C2 OGD/R experiments.

**FIGURE 4:**
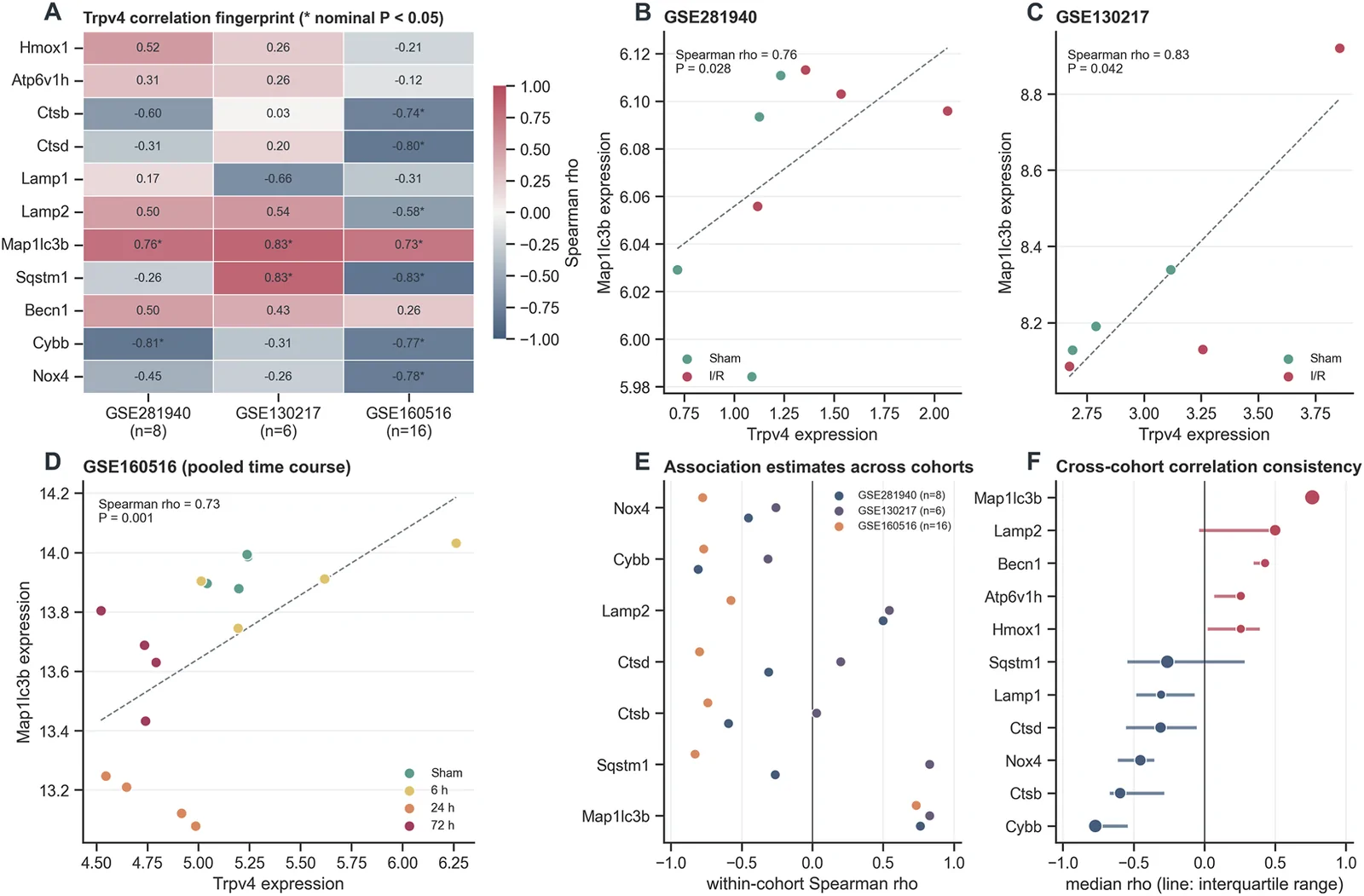
Within-cohort TRPV4 associations with oxidative-response, lysosomal, and autophagy-related genes. **(A)** Correlations between *Trpv4* and prespecified oxidative-response, lysosomal, and autophagy-related genes; asterisks denote nominal Spearman P < 0.05. **(B–D)** Within-cohort *Trpv4*–*Map1lc3b* scatter plots for GSE281940, GSE130217, and the pooled GSE160516 time course. **(E)** Cohort-specific correlation estimates for selected genes. **(F)** Cross-cohort median correlations with interquartile ranges; point size reflects the number of cohorts with nominal P < 0.05. Correlations are descriptive and do not establish causality.

### 4.5 OGD/R upregulates TRPV4 mRNA expression and enhances TRPV4-Associated Ca^2+^ signaling in H9C2 cardiomyoblasts

To experimentally examine the bioinformatics findings, we established an OGD/R model in H9C2 cardiomyoblasts. Cells were exposed to 6 h of OGD followed by 6, 12, or 24 h of reoxygenation. CCK-8 and LDH assays showed a gradual decrease in cell viability and an increase in cell injury with prolonged reoxygenation (Figures 5A,B). Compared with the control group, cell viability was significantly reduced at 12 h (*P* < 0.0001) and 24 h (*P* < 0.0001), while LDH release was increased at the corresponding time points (*P* < 0.0001 and *P* < 0.0001, respectively). No significant difference in cell viability and LDH release was observed between 12 and 24 h (*P* = 0.9926 and *P* = 0.7615, respectively). We therefore selected 12 h for the subsequent experiments, as clear injury was already present while sufficient viable cells remained for pharmacological intervention.

RT-qPCR showed that TRPV4 mRNA expression was increased after OGD/R compared with control cells (*P* = 0.0003; Figure 5C), consistent with the transcriptomic findings. Fluo-4 AM imaging also showed higher intracellular Ca^2+^ levels after OGD/R (*P* = 0.0006 vs. Control), which were reduced by HC-067047 treatment (*P* = 0.0168 vs. OGD/R; Figures 5D,E). These results support the involvement of TRPV4 in OGD/R-associated intracellular Ca^2+^ accumulation in H9C2 cardiomyoblasts.

**FIGURE 5:**
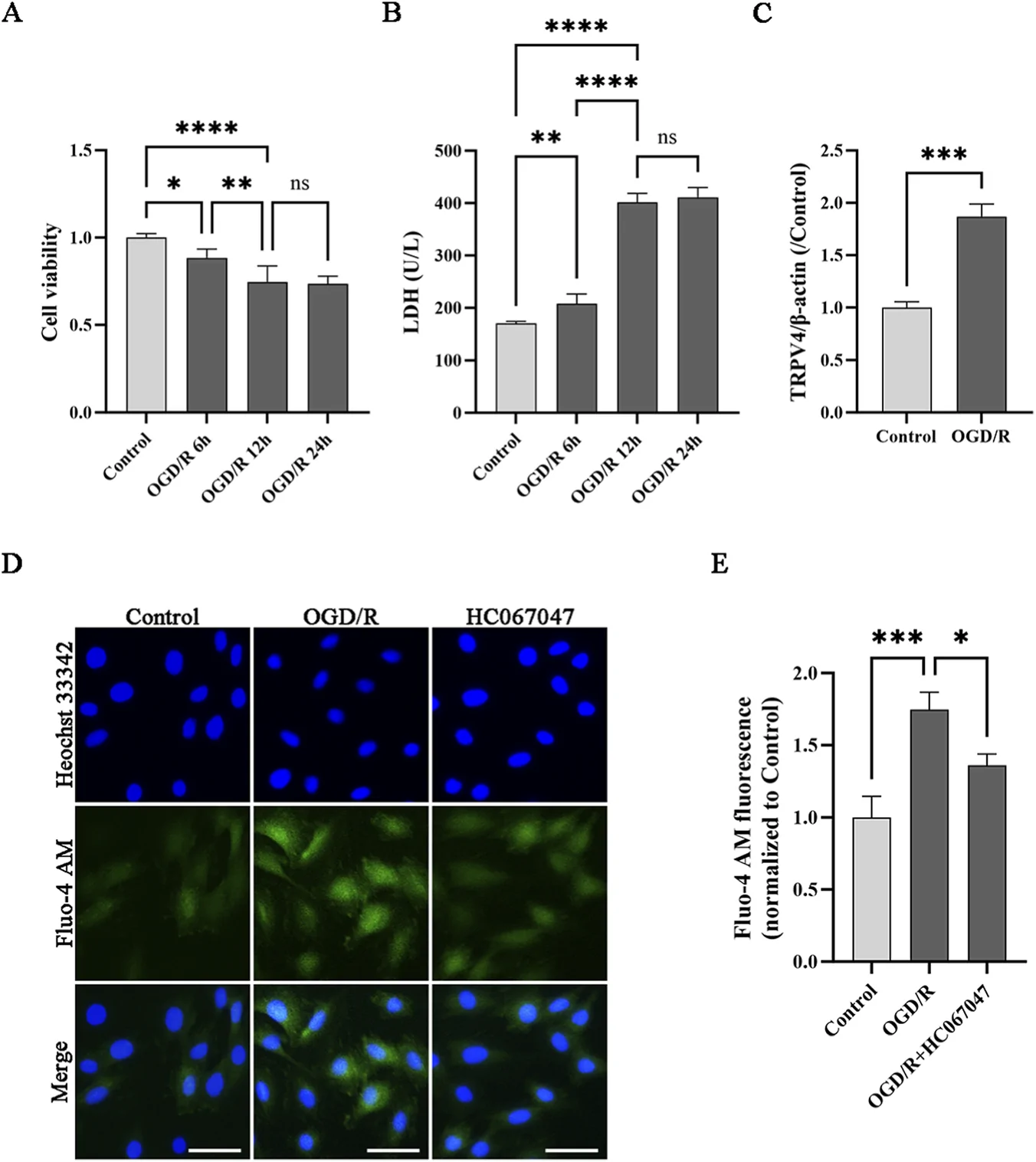
OGD/R increases TRPV4 expression and TRPV4-sensitive Ca^2+^ signaling in H9C2 cardiomyoblasts. **(A)** Cell viability (CCK-8) at 0, 6, 12, and 24 h reoxygenation (n = 5). **(B)** LDH release at corresponding time points (n = 5). **(C)** Relative TRPV4 mRNA expression in H9C2 cells subjected to Control or OGD/R conditions, normalized to β-actin. **(D,E)** Intracellular Ca^2+^ ([Ca^2+^]i) by Fluo-4 AM imaging and quantification (n = 3, scale bar = 50 μm). *P < 0.05, **P < 0.01, ***P < 0.001, ****P < 0.0001.

### 4.6 TRPV4 inhibition attenuates OGD/R-induced lysosomal membrane permeabilization

Having established OGD/R-induced TRPV4 mRNA and Ca^2+^ responses, we next examined whether pharmacological modulation of TRPV4 altered lysosomal membrane integrity. HC-067047 significantly improved cell viability and reduced LDH release (P = 0.0103 for viability and P < 0.0001 for LDH vs. OGD/R), whereas the TRPV4 agonist GSK1016790A further aggravated OGD/R-induced injury (P = 0.0397 and P = 0.0011, respectively; Figures 6A,B). CTSD immunofluorescence showed a punctate lysosomal distribution in control cells that became diffusely cytoplasmic after OGD/R (P < 0.0001 vs. Control), consistent with lysosomal membrane permeabilization and CTSD release. HC-067047 attenuated this redistribution (P < 0.0001 vs. OGD/R), whereas GSK1016790A enhanced it (P = 0.0393 vs. OGD/R; Figures 6C,D). LysoTracker staining similarly showed impaired lysosomal acidification after OGD/R (P < 0.0001 vs. Control); fluorescence was preserved by HC-067047 (P = 0.0051 vs. OGD/R) and further reduced by GSK1016790A (P = 0.0283 vs. OGD/R; Figures 6E,F). These reciprocal antagonist and agonist effects establish a functional link between TRPV4 activity and lysosomal membrane permeabilization and identify TRPV4 as a pharmacologically tractable regulator of OGD/R-induced lysosomal injury.

**FIGURE 6:**
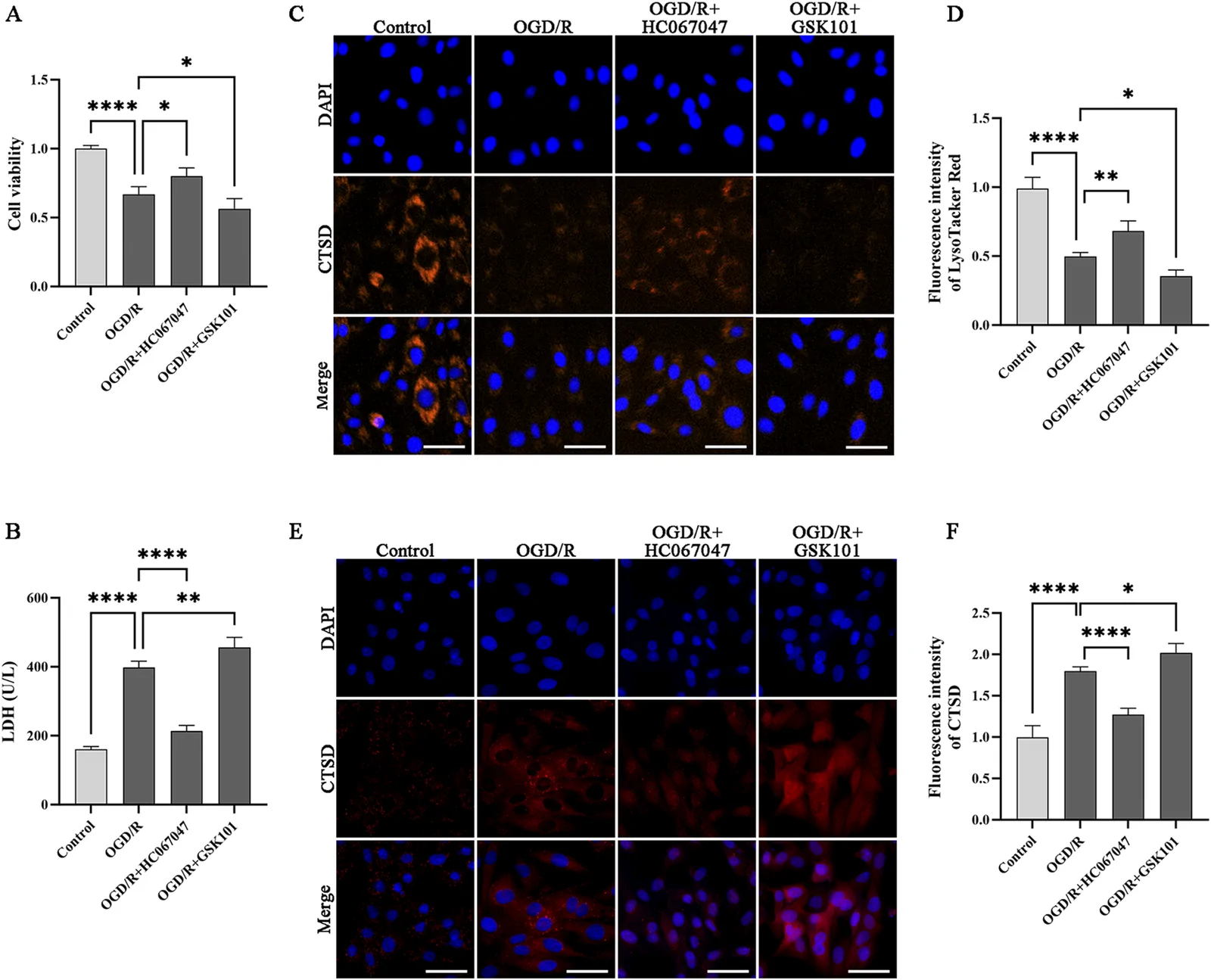
Pharmacological modulation of TRPV4 regulates OGD/R-induced lysosomal membrane permeabilization. **(A)** Cell viability (CCK-8, n = 5). **(B)** LDH release (n = 5). **(C,D)** CTSD immunofluorescence and quantification as LMP readout (n = 3, scale bar = 50 μm). **(E,F)** LysoTracker staining and quantification of lysosomal integrity (n = 3, scale bar = 50 μm). *P < 0.05, **P < 0.01, ***P < 0.001, ****P < 0.0001.

### 4.7 TRPV4 promotes ROS-dependent LMP and inflammatory responses

The prespecified expression-module scores (Figure 2E) and the TRPV4 correlation fingerprint (Figure 4A) linked TRPV4 to oxidative, lysosomal, and autophagy-associated readouts in the transcriptomic disease context. We therefore examined the effect of TRPV4 modulation on intracellular ROS. DCFH-DA staining showed that OGD/R increased ROS levels compared with the control group (P < 0.0001). HC-067047 reduced ROS accumulation (P = 0.0159 vs. OGD/R), whereas GSK1016790A further increased it (P = 0.0028 vs. OGD/R). The increase induced by GSK1016790A was attenuated by NAC treatment (P < 0.0001 vs. OGD/R + GSK1016790A; Figures 7A,B). We next assessed whether these changes were accompanied by an inflammatory response. OGD/R increased the levels of IL-1β, IL-6, and TNF-α in the culture supernatant compared with control cells (all P < 0.0001). HC-067047 reduced the levels of all three cytokines (P < 0.0001, P = 0.0004, and P < 0.0001 vs. OGD/R), whereas GSK1016790A further increased them (P = 0.0339, P = 0.0194, and P = 0.043 vs. OGD/R). NAC attenuated the GSK1016790A-induced increases in IL-1β, IL-6, and TNF-α (all P < 0.0001 vs. OGD/R + GSK1016790A; Figures 7C–E). These findings indicate that TRPV4-associated ROS accumulation is accompanied by an enhanced inflammatory response under OGD/R conditions. We then examined whether ROS was involved in TRPV4-related lysosomal membrane damage. OGD/R reduced LysoTracker fluorescence and increased CTSD redistribution compared with control cells (both P < 0.0001). HC-067047 and NAC attenuated these changes (P = 0.0015 and P < 0.0001 (CTSD), P = 0.0014 and P < 0.0001 (LysoTracker) vs. OGD/R). To further examine the role of ROS, cells treated with HC-067047 were co-treated with t-BHP. t-BHP partially reversed the effects of HC-067047 on LysoTracker fluorescence and CTSD distribution (both P < 0.0001 vs. OGD/R + HC-067047; Figure 8). These results support the involvement of ROS in TRPV4-associated LMP during OGD/R.

**FIGURE 7:**
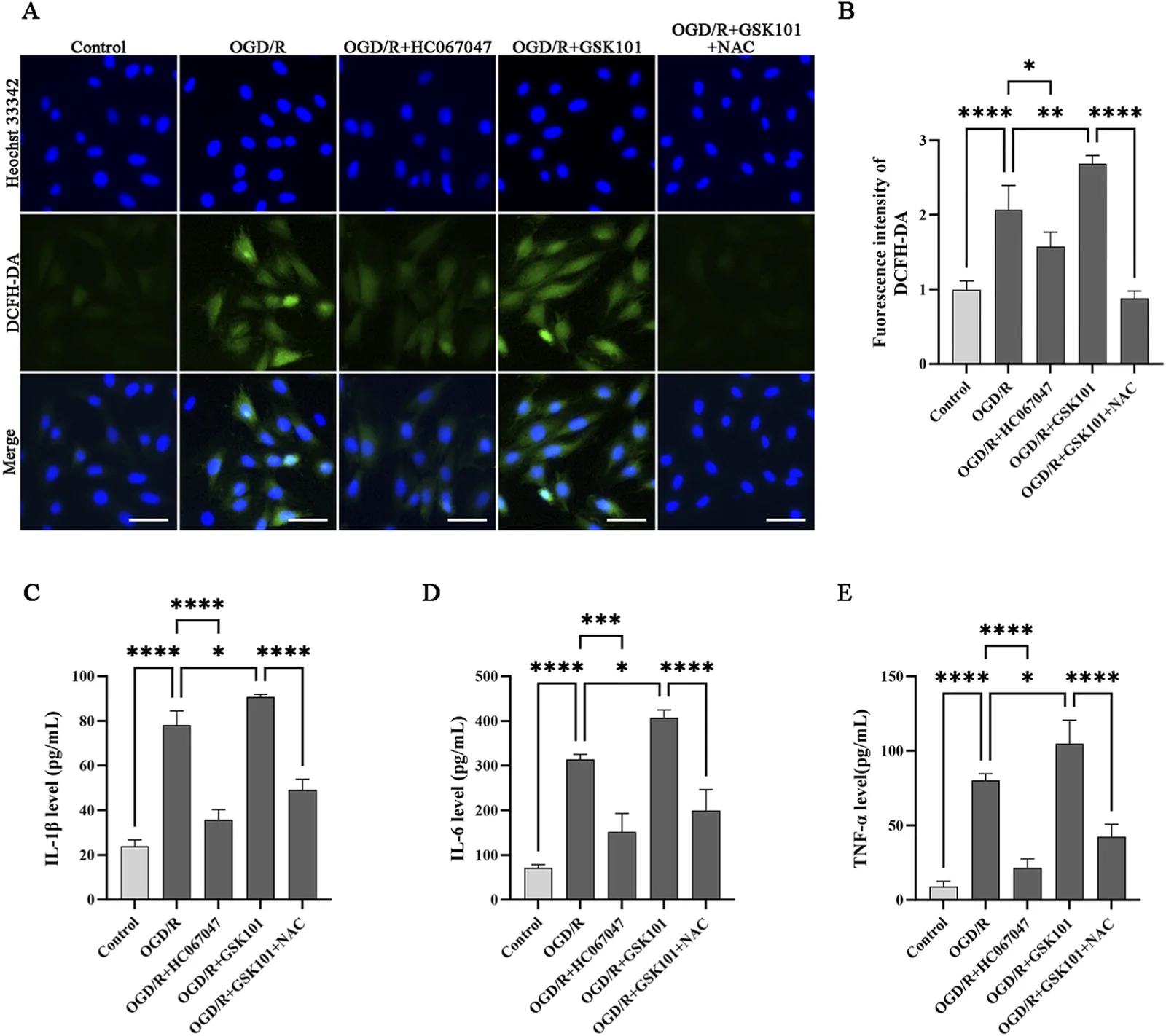
TRPV4 Modulation Alters ROS Accumulation and Inflammatory Responses in OGD/R-Treated H9C2 Cardiomyoblasts. **(A,B)** DCFH-DA staining and quantification of intracellular ROS (n = 3, scale bar = 50 μm). **(C–E)** Levels of IL-1β, IL-6, and TNF-α in the culture supernatant measured by ELISA (n = 5). *P < 0.05, **P < 0.01, ***P < 0.001, ****P < 0.0001.

**FIGURE 8:**
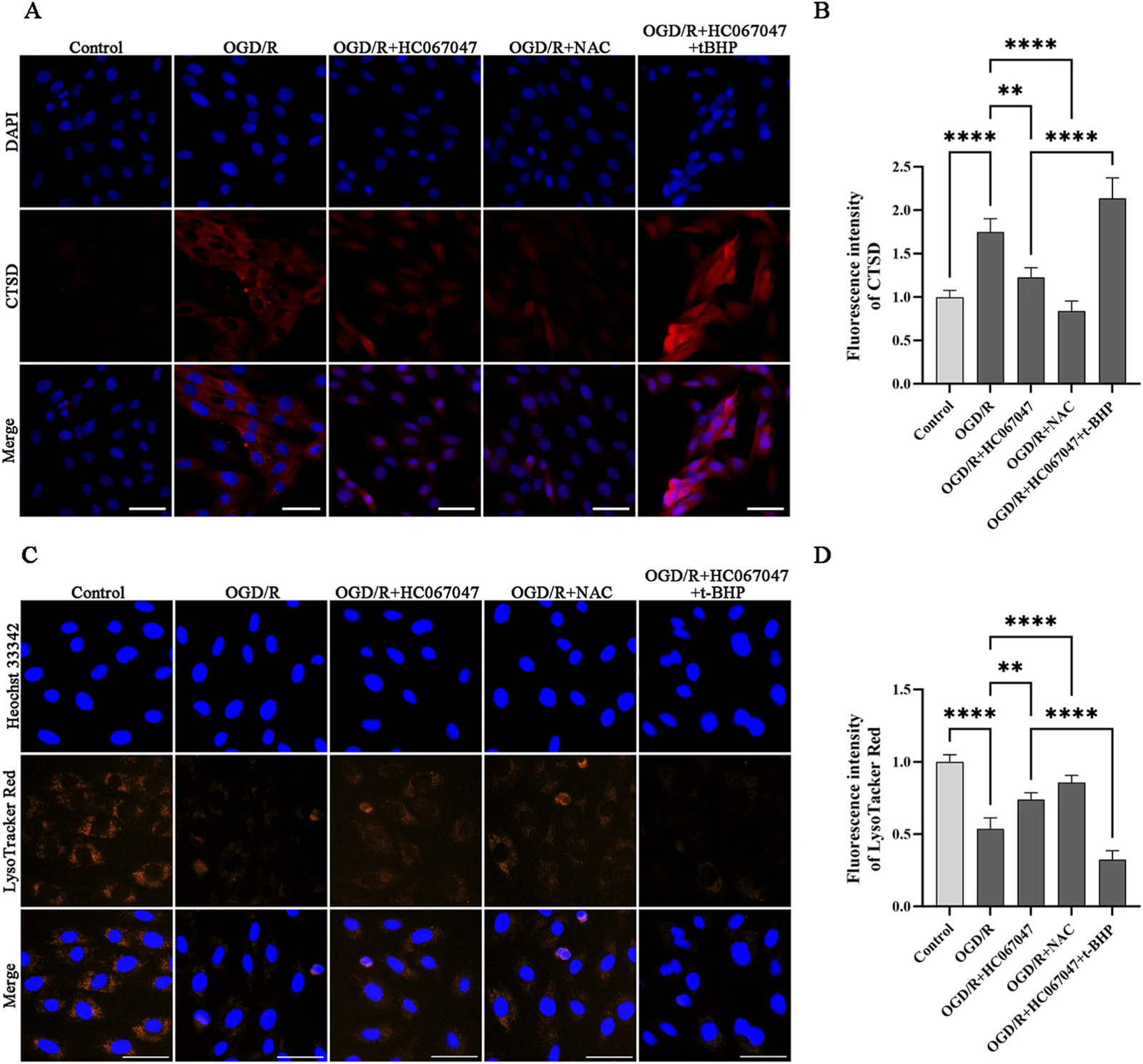
ROS Contributes to TRPV4-Associated Lysosomal Membrane Permeabilization during OGD/R. **(A,B)** CTSD immunofluorescence and quantification (n = 3, scale bar = 50 μm). **(C,D)** LysoTracker staining and quantification (n = 3, scale bar = 50 μm). *P < 0.05, **P < 0.01, ***P < 0.001, ****P < 0.0001.

### 4.8 Lysosomal membrane integrity is associated with autophagic flux during OGD/R

The prespecified module scores (Figure 2E) and cross-cohort expression-module analysis (Figure 3E) provided a transcriptomic context for oxidative, lysosomal, and autophagy-related stress in MI/R. Autophagic flux was therefore examined directly using a chloroquine-based assay. In the absence of CQ, OGD/R increased LC3B puncta and p62 accumulation compared with control cells (both P < 0.0001). CQ caused a further increase in LC3B accumulation in control cells (P < 0.0001 vs. Control), whereas the additional increase was less pronounced after OGD/R (P = 0.8248 for OGD/R + CQ vs. OGD/R; Figures 9A–D). Together with the increase in p62, this pattern was consistent with impaired autophagic degradation under OGD/R conditions.

**FIGURE 9:**
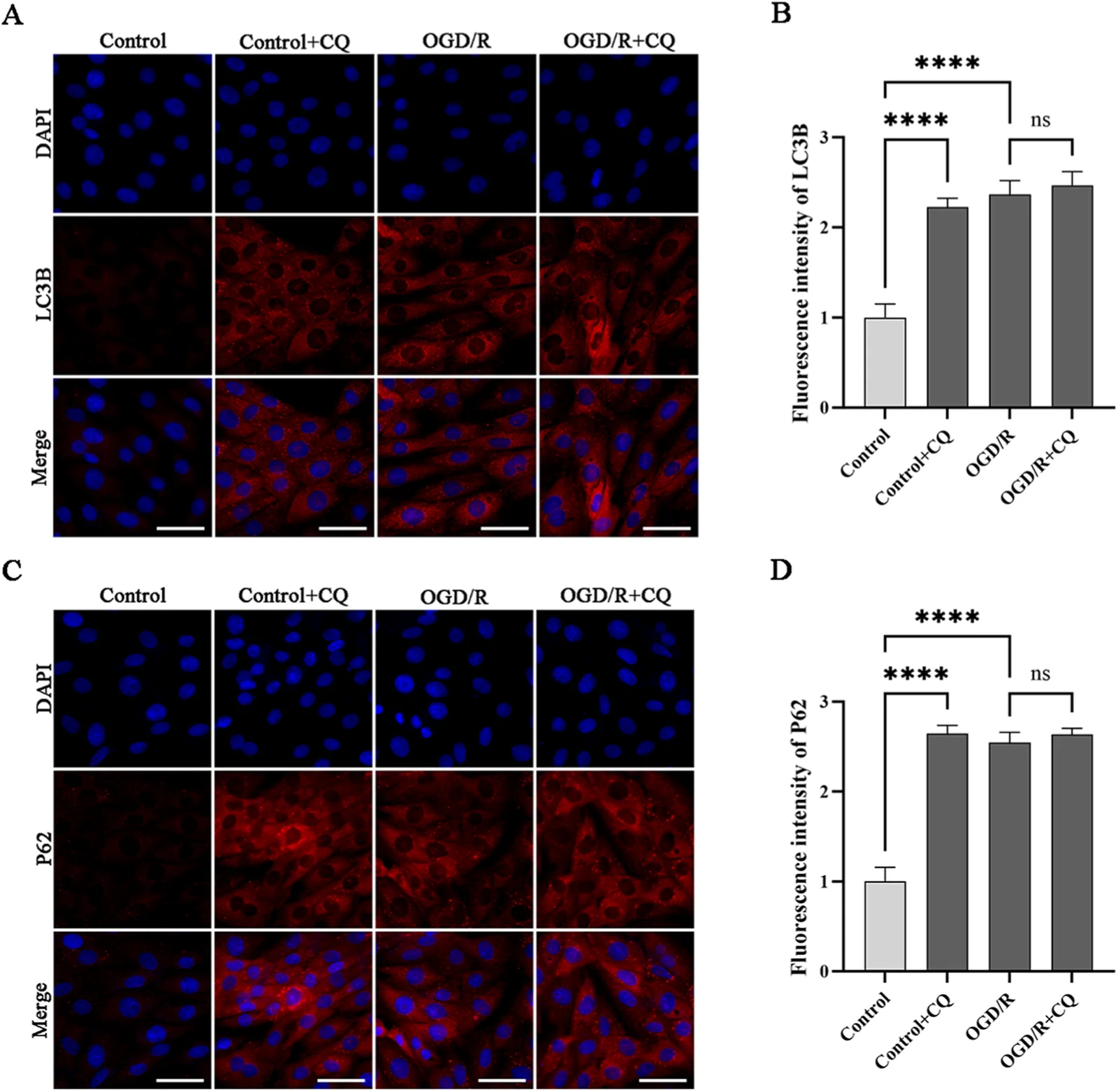
OGD/R is associated with impaired autophagic degradation in a CQ-based immunofluorescence assay. **(A,B)** LC3B immunofluorescence and quantification in control and OGD/R-treated cells with or without CQ (n = 3, scale bar = 50 μm). **(C,D)** p62 immunofluorescence and quantification in control and OGD/R-treated cells with or without CQ (n = 3, scale bar = 50 μm). *P < 0.05, **P < 0.01, ***P < 0.001, ****P < 0.0001.

We then examined whether these autophagy-related changes were associated with lysosomal injury. HC-067047 reduced CTSD redistribution and increased LysoTracker fluorescence compared with OGD/R alone (both P < 0.0001). Mannose, which has previously been reported to preserve lysosomal integrity under cellular stress (Dong et al., 2022), produced similar changes (P < 0.0001 for CTSD and P = 0.0128 for LysoTracker vs. OGD/R; Figures 10A–D). In contrast, LLOMe induced CTSD redistribution and reduced LysoTracker fluorescence in control cells (both P < 0.0001).

**FIGURE 10:**
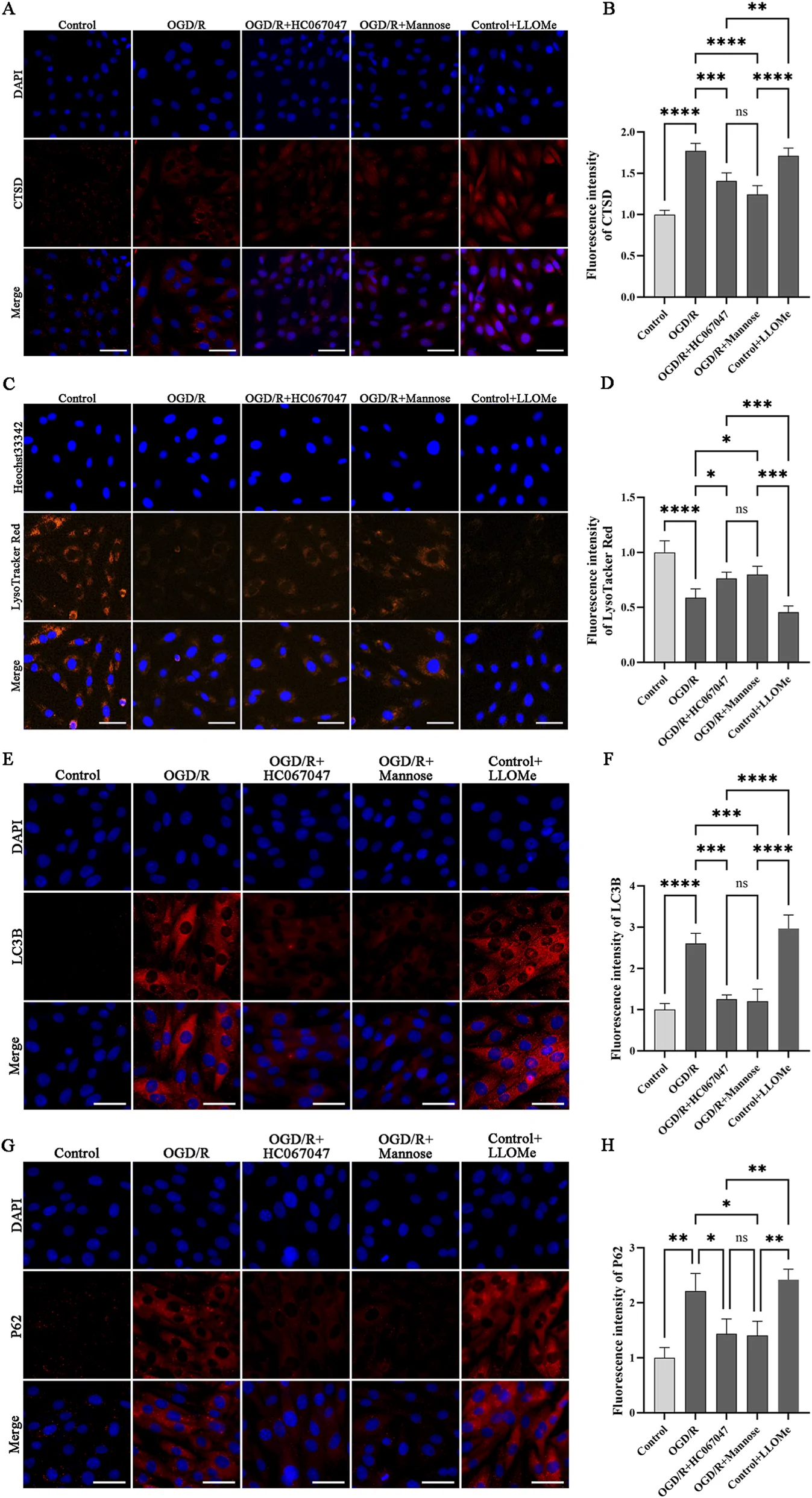
Lysosomal membrane integrity is required for preservation of autophagic flux during OGD/R. **(A,B)** CTSD immunofluorescence and quantification (n = 3, scale bar = 50 μm). **(C,D)** LysoTracker staining and quantification (n = 3, scale bar = 50 μm). **(E,F)** LC3B immunofluorescence and quantification (n = 3, scale bar = 50 μm). **(G,H)** p62 immunofluorescence and quantification (n = 3, scale bar = 50 μm). *P < 0.05, **P < 0.01, ***P < 0.001, ****P < 0.0001.

HC-067047 and mannose also reduced LC3B and p62 accumulation after OGD/R (P < 0.0001 for LC3B and P = 0.0011 for p62 vs. OGD/R), whereas LLOMe increased both markers in control cells (P < 0.0001 for LC3B and P = 0.0003 for p62; Figures 10E–H). These findings suggest that lysosomal injury is associated with impaired autophagic degradation during OGD/R and that preservation of lysosomal function may lessen these autophagy-related changes.

### 4.9 TRPV4 inhibition alleviates autophagic flux impairment through the ROS/LMP pathway

To further examine the relationship between TRPV4, ROS, and autophagy-related changes, we assessed LC3B and p62 by immunofluorescence after TRPV4 inhibition with or without t-BHP treatment. OGD/R increased LC3B and p62 accumulation compared with control cells (P = 0.0001 and P = 0.0049, respectively). HC-067047 reduced both LC3B and p62 accumulation (P = 0.0005 and P = 0.0271 vs. OGD/R; Figures 11A–D). When t-BHP was added together with HC-067047, these effects were partially reversed, with LC3B and p62 levels increasing again (P = 0.0015 and P = 0.0068 vs. OGD/R + HC-067047). Together with the ROS and lysosomal findings described above, these results support the involvement of ROS in the TRPV4-associated autophagy-related abnormalities observed under OGD/R conditions. They are consistent with a model in which TRPV4-related ROS accumulation contributes to lysosomal injury and impaired autophagic degradation.

**FIGURE 11:**
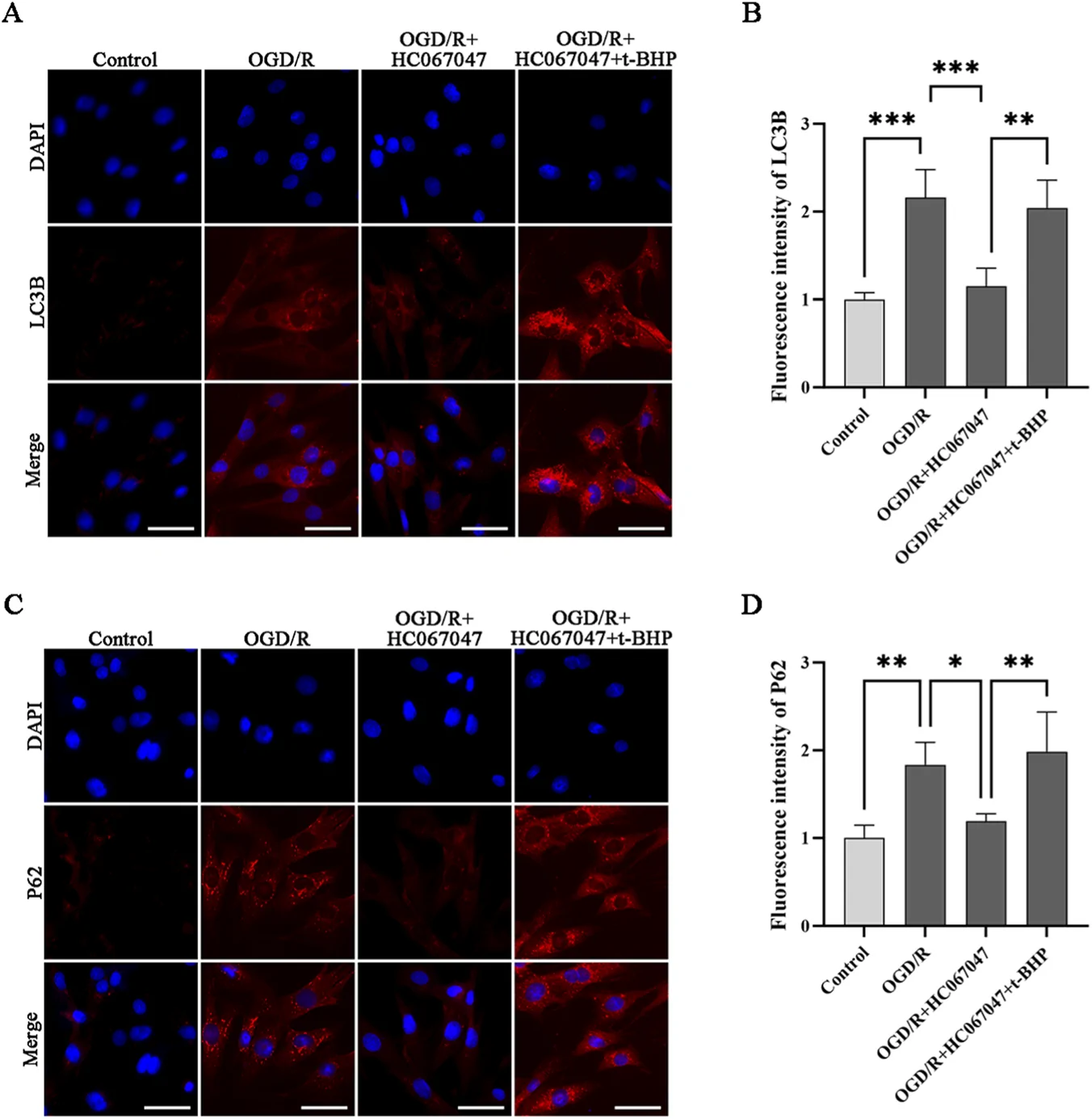
TRPV4 inhibition alleviates autophagic flux impairment through the ROS/LMP pathway in H9C2 cardiomyoblasts. **(A,B)** LC3B immunofluorescence and quantification (n = 3, scale bar = 50 μm). **(C,D)** p62 immunofluorescence and quantification (n = 3, scale bar = 50 μm). *P < 0.05, **P < 0.01, ***P < 0.001, ****P < 0.0001.

## 5 Discussion

This study links myocardial I/R transcriptomic states with TRPV4-dependent cellular phenotypes. Across three disease-matched mouse-heart cohorts, the dominant response comprised conserved oxidative-stress signals and temporally heterogeneous lysosomal and autophagy transcripts. These findings align with established roles of mitochondrial ROS in reperfusion and with evidence that autophagy changes across ischemic and reperfusion stages (Bugg et al., 2020; Aljakna Khan and Sabatasso, 2025; Peng et al., 2024; Mat et al., 2007; Gatica et al., 2015). Impaired lysosomal clearance and autophagic flux are themselves determinants of cardiomyocyte injury (Xing et al., 2022; Xie et al., 2021). The combination of context-dependent TRPV4 expression and a reproducible TRPV4-Map1lc3b correlation supports a pathway-coupling model in which TRPV4 remains biologically relevant without uniform transcriptional induction.

TRPV4 is a non-selective Ca^2+^-permeable cation channel expressed in cardiovascular tissues and has been implicated in myocardial ischemia/reperfusion injury (Sun et al., 2022; Aits and Jaattela, 2013). Our transcriptomic analyses suggest that TRPV4 is not uniformly induced after myocardial I/R, but varies with reperfusion time and experimental context, whereas Hmox1 and several lysosomal-related genes show more consistent changes. This temporal dependence is in line with previous studies showing that autophagy during ischemia/reperfusion is strongly influenced by the stage of injury and the efficiency of lysosomal clearance (Mat et al., 2007; Xing et al., 2022; Yang et al., 2018). Despite this variability, TRPV4 was positively associated with Map1lc3b across all three cohorts. In H9C2 cells, OGD/R increased TRPV4 mRNA expression and intracellular Ca^2+^ levels, while HC-067047 reduced ROS accumulation, lysosomal damage, and cell injury; the opposite changes were observed with GSK1016790A, and NAC attenuated the effects of TRPV4 activation. Together with the changes in IL-1β, IL-6, and TNF-α release, these findings suggest that TRPV4 contributes to OGD/R-induced cardiomyoblast injury through a ROS-dependent lysosomal pathway, with accompanying autophagy-related and inflammatory changes.

LMP is increasingly recognized as an important event in cellular injury and death. Loss of lysosomal membrane integrity allows lysosomal contents, including cathepsins, to redistribute into the cytoplasm, where they can participate in downstream injury signaling (Aits and Jaattela, 2013; Wang et al., 2018; Johansson et al., 2010). ROS is among the best-characterized triggers of LMP, in part because lysosomes contain redox-active iron and have relatively limited antioxidant capacity (Wang et al., 2018; Terman and Kurz, 2013). Excessive ROS generation is also a major feature of myocardial I/R injury (Bugg et al., 2020), and previous studies have linked TRPV4 activation to increased intracellular ROS production (Sun et al., 2022; Bubolz et al., 2012). In our experiments, HC-067047 reduced OGD/R-induced ROS accumulation, while NAC suppressed the increase produced by GSK1016790A. Both HC-067047 and NAC also preserved LysoTracker fluorescence and reduced CTSD redistribution. Conversely, t-BHP partially reversed the protective effects of HC-067047 on these lysosomal readouts. These observations support the involvement of ROS in the relationship between TRPV4 activation and LMP during OGD/R.

LMP may also provide a link between oxidative stress and defective autophagic degradation (Qi et al., 2024; Vietri et al., 2025; He et al., 2023; Repnik et al., 2014). Autophagy is a dynamic process that requires not only autophagosome formation but also autophagosome-lysosome fusion and lysosomal degradation (Shatz and Elazar, 2024). In this study, OGD/R increased both LC3B and p62 levels, suggesting autophagosome accumulation and impaired substrate degradation. CQ-based flux analysis further showed that the CQ-induced increase in LC3B was reduced after OGD/R, indicating that OGD/R impaired autophagic flux at the degradation stage. Previous studies have suggested that TRPV4 is involved in the regulation of autophagy. In colon cancer cells, inhibition or silencing of TRPV4 induced autophagy through suppression of the AKT/mTOR pathway, suggesting that TRPV4 activity can influence autophagy-related signaling (Liu et al., 2019). In hepatic stellate cells, TRPV4 was also reported to regulate autophagy-dependent cell survival and apoptosis-related responses, further supporting a functional connection between TRPV4 signaling and autophagy (Zhan et al., 2015). More recently, inhibition of TRPV4 was shown to improve α-synuclein degradation by restoring the autophagy-lysosomal pathway in an MPP + -induced cellular model of Parkinson’s disease (Bai et al., 2025). Our findings extend these observations to an OGD/R cardiomyoblast model. HC-067047 and mannose, which has previously been reported to preserve lysosomal integrity under cellular stress, reduced CTSD redistribution and improved LysoTracker fluorescence after OGD/R. Both treatments were also associated with lower LC3B and p62 accumulation, whereas LLOMe produced the opposite pattern in control cells. In addition, t-BHP partially reversed the effects of HC-067047 on LC3B and p62 accumulation. These results are consistent with a model in which TRPV4-associated ROS accumulation promotes lysosomal injury and contributes to impaired autophagic degradation.

Overall, our data connect TRPV4-associated Ca^2+^ and ROS changes with lysosomal membrane integrity, autophagy-related degradation, and inflammatory responses during OGD/R. The transcriptomic findings also indicate that TRPV4 is better viewed as a context-dependent component of the myocardial stress response than as a uniformly induced marker. This distinction suggests that the contribution of TRPV4 may depend on cell state and functional activation rather than on large changes in bulk transcript abundance alone. Further work in primary cardiomyocytes and *in vivo* MI/R models should clarify how TRPV4 activity, ROS production, LMP, autophagic degradation, and inflammatory signaling change over the course of reperfusion and which myocardial cell populations are most responsive to TRPV4 modulation.

### 5.1 Limitation

This study has several limitations. The mechanistic experiments were performed in H9C2 rat cardiomyoblasts, a widely used *in vitro* model of ischemia/reperfusion-related injury. However, H9C2 cells do not fully reproduce the electrophysiological, metabolic, contractile, and structural properties of mature cardiomyocytes. Therefore, the present findings should primarily be interpreted within the OGD/R-treated H9C2 model. Further studies using primary or human cardiomyocyte models and *in vivo* MI/R models will be useful to determine the physiological relevance of the TRPV4–ROS–LMP pathway. Autophagy-related changes were assessed by LC3B and p62 immunofluorescence together with CQ-based flux analysis. The reduced additional accumulation of LC3B after CQ treatment under OGD/R conditions, together with p62 accumulation, provided evidence consistent with impaired autophagic degradation. Because LC3B immunofluorescence does not distinguish LC3-I from LC3-II, these findings were interpreted as supportive rather than definitive evidence of altered autophagic flux. Future studies combining immunoblotting or tandem fluorescent LC3 reporters may provide additional confirmation of these observations.

## 6 Conclusion

Disease-matched mouse-heart transcriptomes revealed consistent oxidative-stress-related changes, dynamic lysosomal and autophagy-related transcription, and a context-dependent TRPV4 response, with a reproducible positive association between TRPV4 and Map1lc3b. In H9C2 cardiomyoblasts, OGD/R increased TRPV4-associated intracellular Ca^2+^ and ROS accumulation, accompanied by lysosomal membrane permeabilization, impaired autophagic degradation, and enhanced inflammatory responses. Together, these findings support the involvement of the TRPV4–ROS–LMP pathway in OGD/R-induced cardiomyoblast injury and provide a basis for further evaluation in primary cardiomyocytes and *in vivo* MI/R models.

## Data Availability

The datasets presented in this study can be found in online repositories. The names of the repository/repositories and accession number(s) can be found in the article/supplementary material.
